# High-affinity nitrate/nitrite transporters NrtA and NrtB of *Aspergillus nidulans* exhibit high specificity and different inhibitor sensitivity

**DOI:** 10.1099/mic.0.000088

**Published:** 2015-07

**Authors:** Naureen Akhtar, Eugenia Karabika, James R. Kinghorn, Anthony D.M. Glass, Shiela E. Unkles, Duncan A. Rouch

**Affiliations:** ^1^​ School of Biology, University of St Andrews, St Andrews, Fife, KY16 9TH, UK; ^2^​ Biochemistry Laboratory, Chemistry Department, University of Ioannina, Ioannina 45110, Greece; ^3^​ Department of Botany, University of British Columbia, Vancouver, British Columbia, V6T 1Z4, Canada; ^4^​ Biotechnology and Environmental Biology, RMIT University, Melbourne, Australia

## Abstract

The NrtA and NrtB nitrate transporters are paralogous members of the major facilitator superfamily in *Aspergillus nidulans*. The availability of loss-of-function mutations allowed individual investigation of the specificity and inhibitor sensitivity of both NrtA and NrtB. In this study, growth response tests were carried out at a growth-limiting concentration of nitrate (1 mM) as the sole nitrogen source, in the presence of a number of potential nitrate analogues at various concentrations, to evaluate their effect on nitrate transport. Both chlorate and chlorite inhibited fungal growth, with chlorite exerting the greater inhibition. The main transporter of nitrate, NrtA, proved to be more sensitive to chlorate than the minor transporter, NrtB. Similarly, the cation caesium was shown to exert differential effects, strongly inhibiting the activity of NrtB, but not NrtA. In contrast, no inhibition of nitrate uptake by NrtA or NrtB transporters was observed in either growth tests or uptake assays in the presence of bicarbonate, formate, malonate or oxalate (sulphite could not be tested in uptake assays owing to its reaction with nitrate), indicating significant specificity of nitrate transport. Kinetic analyses of nitrate uptake revealed that both chlorate and chlorite inhibited NrtA competitively, while these same inhibitors inhibited NrtB in a non-competitive fashion. The caesium ion appeared to inhibit NrtA in a non-competitive fashion, while NrtB was inhibited uncompetitively. The results provide further evidence of the distinctly different characteristics as well as the high specificity of nitrate uptake by these two transporters.

## Introduction

Nitrate is a major nitrogen source for most micro-organisms and plants. The transport of nitrate (and nitrite) by most bacteria, algae, fungi and plants across membrane barriers is carried out mainly by high-affinity transporters (Trueman *et al.*, 1996). Such high-affinity transporters belong to the nitrate/nitrite porter family, NNP: TC 2.A.1.8 (www.tcdb.org; Pao *et al.*, 1998; Forde, 2000), a subfamily forming a distinct cluster of the largest secondary transporter family, the major facilitator superfamily (MFS: TC 2.A.1). Most eukaryotes express several versions of Nrt transport proteins, encoded by individual genes. For instance, higher plants including crop plants have numerous copies, e.g. *Arabidopsis thaliana* has seven members of the *NRT2* gene subfamily and multiple copies of the *NRT1* subfamily (Okamoto *et al.*, 2003), the latter group belonging to the related MFS family of proton-dependent oligopeptide transporter family (POT:TC 2.A.17). The lower eukaryote *Aspergillus nidulans* possesses two nitrate transporters, NrtA and NrtB, which share 61 % sequence identity (Unkles *et al.*, 2001). Nitrate transport in a WT strain of *A. nidulans* has been shown to be proton-dependent (Downey & Gedeon, 1994), and transport of nitrate by NrtA has been shown specifically to be energized by protons (Boyd *et al.*, 2003).

The precise functional role of individual Nrt paralogues has not yet been clarified, and assessing the structures was until recently intractable to crystallographic approaches. Notwithstanding this impasse, biochemical, genetic and physiological methods have provided valuable experimental information (Unkles *et al.*, 2001, 2011, 2012; Wang *et al.*, 2008). Recently, however, the crystal structure of the *Escherichia coli* nitrate/nitrite exchanger, NarK, a member of the NNP family like NrtA and NrtB, has been reported by Zheng *et al.* (2013). Functionally crucial residues in both NrtA and NrtB, as identified previously by mutagenesis and functional studies, are identical to those reported in the NarK crystal structure. The NarK structure can now be used conveniently as a model to understand the structure and function of *A. nidulans* NrtA and NrtB as well as other nitrate transporters. In addition, the crystal structure of the bacterial nitrate transporter NarU, also a member of the NNP family, has been reported (Yan *et al.*, 2013). The structure of the NRT1.1 nitrate transporter of *Arabidopsis* (also called CHL1 or NPF6.3) has been solved to 3.25 Å resolution (Sun *et al.*, 2014). This was the first nitrate transporter identified in higher plants and belongs to the NRT1 family.

Given that cellular organisms generally have more than one nitrate transporter it is likely these vary in transport properties, to ensure optimal uptake of nitrate under different environmental or physiological conditions. For example, NrtA shows a lower affinity for nitrate compared with NrtB, with *K*_m_ values of 96 and 11 μM, respectively. The NrtB transporter thus might be more effective at scavenging nitrate from low external concentrations, for which the lower affinity of the NrtA protein would be less effective (Unkles *et al.*, 2001). Interestingly, the *Arabidopsis* NRT1.1 transporter has two distinct affinity modes, which may be switched by shifts between monomer and dimer structures of the transport proteins, under control by specific phosphorylation of the transporter (Sun *et al.*, 2014).

A further important variable is the range of substrates recognized by transporters. Both NrtA and NrtB transporters also provide efficient uptake of the structurally similar compound nitrite, with *K*_m_ values of 16 and 11 μM, respectively (Wang *et al.*, 2008). In terms of substrate specificity NarU showed reduced transport of two ionic compounds, phosphate and ammonia, compared with nitrate (Yan *et al.*, 2013), while there have been no reports on substrate specificity for NarK.

One way to further assess substrate specificity is to examine inhibition of transport by toxic chemicals. In *Aspergillus*, inhibition of nitrate transport by chlorate has been studied by several research groups (Brownlee & Arst, 1983; Unkles *et al.*, 1991, 2001; Siddiqi *et al.*, 1992; Kosola & Bloom, 1996; Kinghorn *et al.*, 2005). However, a drawback of these earlier studies has been that the data have often been obtained from (i) organisms with a number of different Nrt nitrate transporters, and therefore the transport process analysed is mediated by more than one nitrate transporter protein (Orsel *et al.*, 2006), (ii) heterologously synthesized Nrt protein, which might not reflect homologously produced protein characteristics (Zhou *et al.*, 2000), or (iii) complex tissue such as the plant root with multiple cell types (Enstone *et al.*, 2002; Baxter *et al.*, 2009). Nevertheless, semiquantitative data for chlorate inhibition of nitrate transport by NrtB (*crnA1* mutant) have been reported (Brownlee & Arst, 1983).

**Fig. 1.**
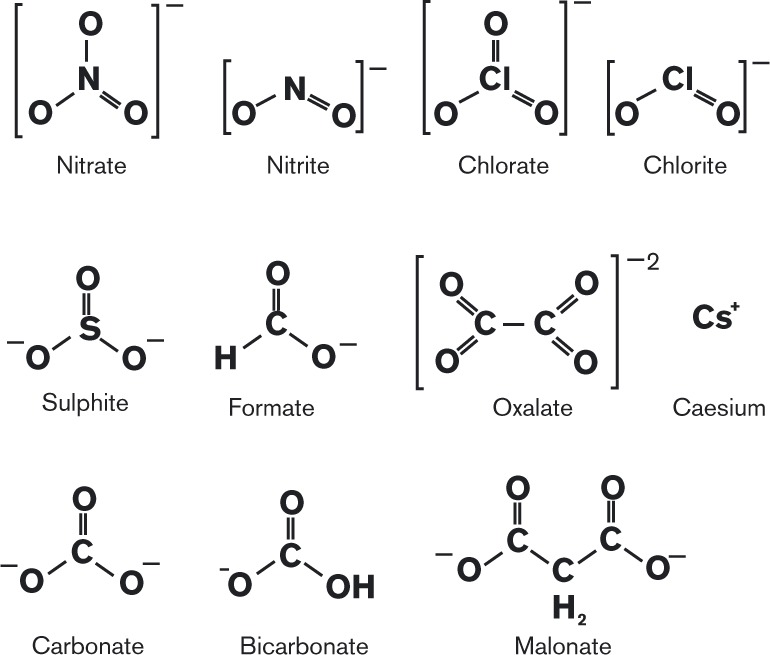
Structure of potential inhibitors of nitrate transport. Chlorate (ClO_3_^− ^) has a trigonal pyramid structure, while nitrate (NO_3_^− ^) is trigonal planar. Sulphite (SO_3_^2 − ^) has the same trigonal pyramid structure as chlorate. The molecular structure of bicarbonate (HCO_3_^− ^), a compound commonly found in environmental water bodies, is also similar to nitrate but has a carbon atom in the centre instead of nitrogen. In the trigonal planer structure of formate (HCOO^− ^), at one position hydrogen is present instead of oxygen as compared with the nitrate molecule. Malonate [CH_2_(COO)_2_^2 − ^] resembles two nitrate molecules joined together, and oxalate [(COO)_2_^2 − ^] differs from malonate in having a shorter carbon chain. Caesium was used in its cationic form, Cs^+^. The above ions were tested for their inhibitory effect on growth of *A. nidulans* in the presence of nitrate as sole N source, and selected anions were assessed for their inhibitory effect on the activity of *A. nidulans* nitrate transporters NrtA and NrtB in nitrate uptake assays. The figure was created using Corel Draw (version 6).

Our aims were (i) to investigate the specificity of nitrate recognition by individual Nrt proteins and (ii) to detect further functional differences between different Nrt proteins. To do this we carried out inhibition studies on *A. nidulans* NrtA and NrtB, employing various genetic mutants. Initially, we employed chlorate as an inhibitor of nitrate transport. Using this approach we also studied other potential inhibitor molecules, which share a similar molecular structure to nitrate. These included the anions bicarbonate, formate and sulphite (Fig. 1). In addition, malonate and oxalate were assessed, which both resemble two nitrate molecules joined together. These compounds were included to address the possibility of multiple nitrate-binding sites being present in NrtA and NrtB, as suggested by the repeated nitrate signature motifs in these proteins (Unkles *et al.*, 2004, 2012).

It has been previously suggested that toxicity of chlorate is due to its reduction to chlorite by nitrate reductase (LaBrie *et al.*, 1991; Siddiqi *et al.*, 1992), or due to chlorite being present as a contaminant of chlorate (Zhou *et al.*, 2000). In addition, chlorate significantly inhibits the activity of nitrate reductase (McDonald & Coddington, 1974). Caesium also is an established growth inhibitor of nitrate uptake-defective strains, in not only *A. nidulans* (Brownlee & Arst, 1983), but also other fungi (Gao-Rubinelli & Marzluf, 2004). Therefore, the effect of chlorite and caesium was also studied in detail.

As well as exhibiting the capability to import nitrate, cells may also export this nitrogen source and related compounds. The yeast *Hansenula polymorpha* (also known as *Ogataea angusta*) possesses three transporters that export nitrate or/and nitrite: Nar1, which exports both nitrate and nitrite, and Ssu1 and Ssu2, which both transport mainly sulphite but also nitrate (Cabrera *et al.*, 2014). Sulphite export has also been reported by Ssu1 from *Aspergillus fumigatus* (Léchenne *et al.*, 2007). Nar1 is a member of the formate–nitrite transporter family (FNT: TC 1.A.16). Similarly, under certain conditions, the *A. nidulans* NrtA transporter can export nitrate and nitrite (Wang *et al.*, 2007), and the *A. nidulans* FNT nitrite transporter, NitA, can export nitrite (Wang *et al.*, 2008).

Results indicated nitrate transport by NrtA and NrtB exhibited different sensitivities to chlorate and caesium, while a range of other chemicals with comparable structures to nitrate had no significant effect on transport. The basis of substrate specificity by NrtA and NrtB is proposed to be recognition by the transporters of both structural similarity to nitrate and the intermediate degree of charge separation between the central atom (N in nitrate and nitrite) and distal O atoms of the substrate.

## Methods

### Fungal strains

The *A. nidulans* strains used in this study are (i) the WT positive growth control (on nitrate) strain, *biA1*, (ii) *nrtA1* (formerly *crnA1*; Brownlee & Arst, 1983) and *nrtA747*, both of which harbour a deletion mutation, resulting in loss of function (these two mutants gave similar results and so the results for mutant *nrtA1* only are given in this article), (iii) knockout, deletion strain *nrtB110* (Unkles *et al.*, 2001) and (iv) the double deletion negative growth control (on nitrate) mutant tbl110 (*nrtA747* *nrtB110)* (Unkles *et al.*, 2001).

### *Escherichia coli* strains, plasmids and media

Standard procedures were used for propagation of plasmids, as well as for subcloning and maintenance of plasmids within *E. coli* strain DH5α.

### Growth tests

Fungal growth tests were carried out in standard Petri dishes containing defined *Aspergillus* minimal medium (AMM) agar (Cove, 1976), with sodium nitrate or proline as the sole nitrogen source, at 1 or 10 mM concentration. Strains were tested for inhibition by bicarbonate, formate, oxalate, chlorite, sulphite (all purchased as the sodium salt), chlorate (potassium salt), malonate (acid form) or caesium (chloride salt). Stock aqueous solutions of inhibitors were adjusted to pH 6.5 and prepared immediately before use. It should be noted that at pH 6.5, bicarbonate is in approximately 50 % equilibrium with carbonic acid. The nitrogen sources and potential inhibitor were added to cool but still molten AMM or liquid AMM (Cove, 1976), to give a final concentration as indicated in the text. Cultures were incubated routinely at 37 °C for 48 h, and the growth response was scored.

### Minimum inhibitory concentration (MIC) tests

**Fig. 2.**
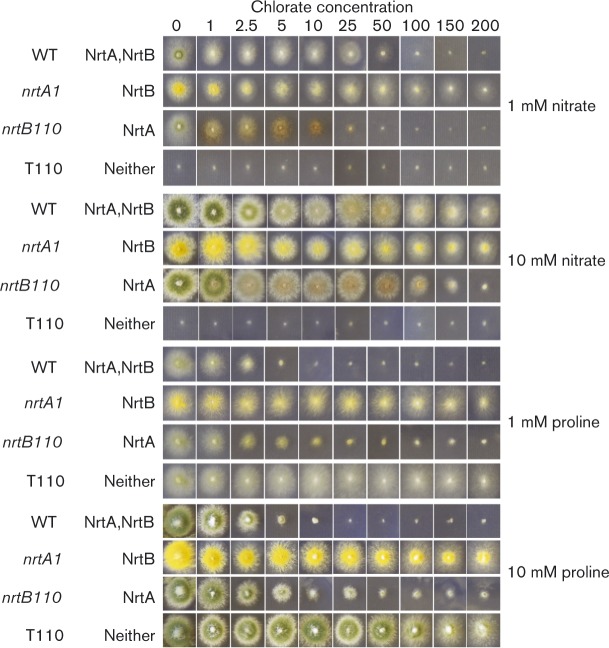
Growth inhibition of the WT and mutant strains by chlorate. Tests of growth inhibition by chlorate were carried out on solid AMM that contained a range of chlorate concentrations, with sole nitrogen source nitrate or proline at 1 or 10 mM concentration.

**Fig. 3.**
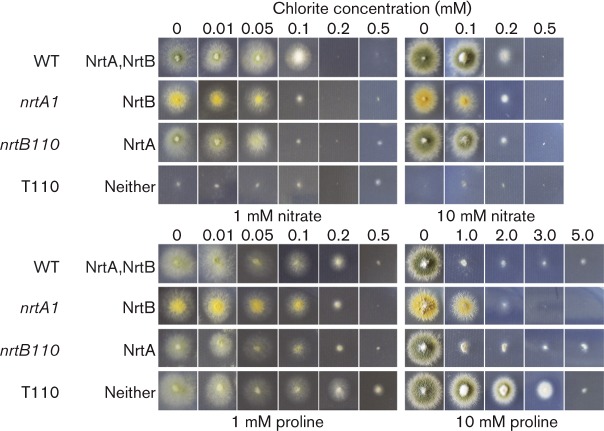
Growth inhibition of the WT and mutant strains by chlorite.

**Fig. 4.**
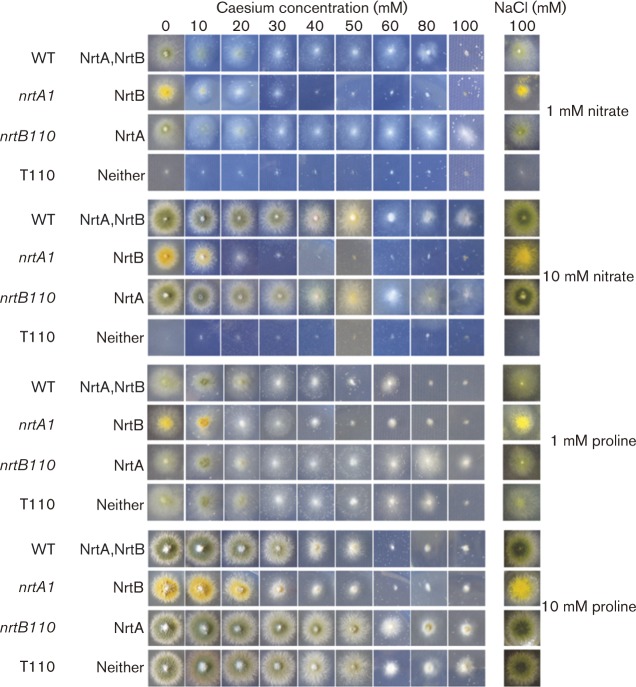
Growth inhibition of the WT and mutant strains by caesium, as a chloride salt. As a control for potential inhibition by chloride, growth on 100 mM sodium chloride is also shown, on the far right.

**Fig. 5.**
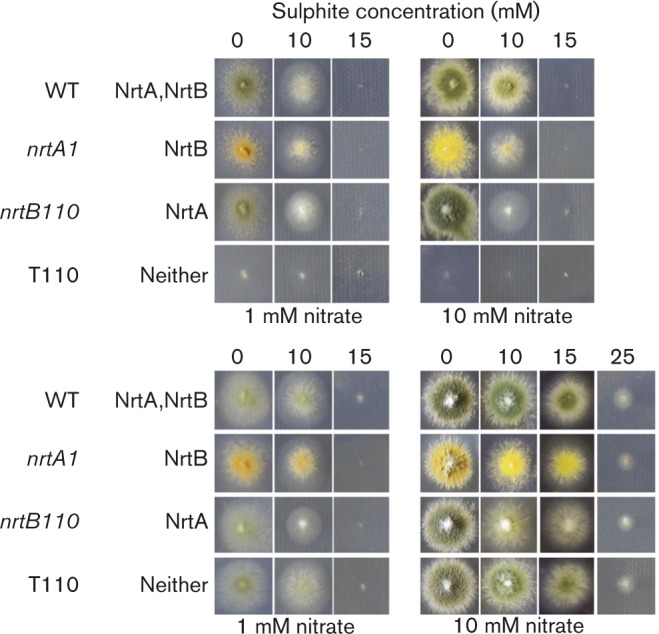
Growth inhibition of the WT and mutant strains by sulphite.

To assess the MIC of chlorate, chlorite, caesium and sulphite, the WT and mutant strains were grown on solid AMM that contained either 1 or 10 mM proline, or 1 or 10 mM nitrate, as the sole nitrogen source and amended with a range of concentrations of each compound: chlorate, 0 to 200 mM; chlorite, 0 to 0.5 mM or 0 to 5.0 mM; caesium, 0 to 100 mM; sulphite, 0 to 25 mM. Each treatment was carried out in five replicates. The MIC was taken as the lowest concentration that exhibited growth equivalent to the baseline appearance of strain tbl110 on nitrate as sole nitrogen source as seen in Figs. 2–5. As caesium was utilized as a chloride salt, a control for potential inhibition by chloride was assessed, by growth on 100 mM sodium chloride (Fig. 4).

### Chemical purity

In this study, 99 % pure grade potassium chlorate (BDH) was used. Sodium chlorite used in our experiments was Alfa Aesar 80 % pure, with 10 % sodium chloride, 3 % sodium chlorate, 3 % sodium hydroxide and 2 % sodium carbonate contamination. Sodium chlorite from other companies had a similar level of contamination. All other potential inhibitors used in this survey were at least 99 % pure grade.

### Net nitrate uptake assays

These were performed on cultures grown in liquid AMM with 5 mM urea as nitrogen source for a total of 6.5 h at 37 °C (Brownlee & Arst, 1983). Urea was used as it (i) is a neutral nitrogen source, neither inducing nor repressing nitrate transport, (ii) has been used routinely in the past and (iii) permitted direct comparison with previously published results. Preliminary results using proline, another neutral nitrogen source, as the sole nitrogen showed no appreciable difference. Transporter activity was induced by the addition of 10 mM sodium nitrate 100 min prior to assay as described previously (Brownlee & Arst, 1983). Assays were carried out in triplicate, generally at pH 6.5, on three independently grown cultures for each strain and expressed as nmol nitrate removed from the medium min^ − 1^ (mg dried mycelium)^ − 1^, as discussed before (Brownlee & Arst, 1983; Kinghorn *et al.*, 2005; Unkles *et al.*, 2012). Both carbonic acid and bicarbonate chemical species can occur at pH 6.5. Thus potential inhibition of nitrate transport by carbonic acid/bicarbonate was tested at pH 6.5 and bicarbonate at pH 8.3. To assess the effect of carbonate, assays were carried out at pH 11.3, but nitrate transport per se was not detectable at that pH value and so this chemical form could not be tested.

To effectively assess net uptake, it is necessary that any export, i.e. simultaneous transport in the reverse direction, is minimal. For a WT strain the rate of nitrate efflux was 8 % of influx (Wang *et al.*, 2007). This rate can be taken as a maximum, as it was assessed by reverse downhill transport, and here nitrate concentrations favoured uptake. Therefore, the use of the net uptake assay method was acceptable for relative comparisons of uptake properties. Data for sulphite assays could not be assessed as, under the assay conditions, sulphite appeared to directly cause the chemical reduction of nitrate in the absence of mycelium. The other chemicals tested did not affect the oxidation state of nitrate within the assay period and at the concentrations used.

### Determination of kinetic parameters

Values of *K*_m_ and *V*_max_ were calculated by linear regression of Lineweaver–Burk analyses (1/*v* against 1/*s*, where *v* is net uptake and *s* is substrate concentration). The kinetic values of nitrate transport by the WT and mutants were compared with those from a previous report (Unkles *et al.*, 2001). For the mutant expressing NrtB both *V*_max_ and *K*_m_ values were statistically similar to the previous data. However, for both the WT and the mutant expressing NrtA, *K*_m_ values were about twofold lower than in the earlier investigation, while the associated *V*_max_ values were statistically similar. These relatively small differences in *K*_m_ values might possibly be due to minor differences in growth conditions between the two studies. For example, it has been established that kinetic values can vary with growth time (Unkles *et al.*, 2001). Therefore the kinetic values in the absence of inhibitors from the present study were used as control values. Each *K*_i_ value was determined by regression of the rates of net nitrate uptake transport versus the range of concentrations of a particular inhibitor: linear or nonlinear regression was used, depending on which type provided the highest *r* ^2^ value. Calculated from each regression was the concentration of the inhibitor that reduced net nitrate uptake rate by 50 % of the control (without inhibitor) rate, which was assessed as the *K*_i_ value. Lineweaver–Burk analyses were carried out to determine the nature of the inhibition of nitrate transport for each inhibitor.

### Sequence comparisons

Protein sequences of Nrt1.1, Nar1, Ssu1 and Ssu2 from *H. polymorpha* and Ssu1 from *A. fumigatus* (GenBank accession numbers: AEE28838, AAY27379, AAX54671, CCQ44061 and AAX54670, respectively) were searched against all proteins encoded by the genome of *A. nidulans*, strain FGSC A4, using the NCBI blastp program (Altschul *et al.*, 1990: http://blast.ncbi.nlm.nih.gov/Blast.cgi?CMD = Web&PAGE_TYPE = BlastHome).

## Results

### Chlorate inhibition

Mutant strain *nrtB110* (expressing NrtA activity) and *nrtA1* and *nrtA747* (expressing NrtB activity) as well as controls, WT (expressing NrtA and NrtB) and the double mutant strain tbl110 (*nrtA747 nrtB110*, devoid of NrtA and NrtB activities) were examined for growth on solid AMM containing 1 mM nitrate as the sole source of nitrogen in the presence of 1 to 200 mM chlorate. The rationale for using 1 mM nitrate is that this nitrate concentration is near the growth-limiting nitrogen concentration, and so the additional presence of inhibitors at excess concentrations would a priori retard growth, by inhibiting nitrate uptake. The negative control strain, tbl110, with 1 mM nitrate or 1 mM proline as the sole source of nitrogen (on solid AMM) failed to grow on nitrate as expected but grew like the WT on proline (Unkles *et al.*, 2001; Kinghorn *et al.*, 2005) (Table 1, Fig. 2). The WT strain and mutant *nrtB110* (NrtA transporter) exhibited growth limitation in the presence of chlorate with MIC values of 100 and 50 mM, respectively, with 1 mM nitrate as sole nitrogen source, while mutant *nrtA1* (NrtB transporter) continued to grow at the highest chlorate concentration tested, 200 mM. In contrast to incubation with 1 mM nitrate, 10 mM nitrate overcame chlorate inhibition with WT and mutant *nrtB110,* as would be expected for a specific inhibitor of nitrate transport: strain *nrtB110* required around 200 mM chlorate to stunt growth completely under this condition (Table 1, Fig. 2). With 1 mM proline as the sole source of nitrogen, the *nrtA1* mutant strain (with functional NrtB) was resistant to chlorate toxicity, even at concentrations as high as 200 mM (Table 1, Fig. 2), whilst the mutant expressing NrtA (*nrtB110*) as well as the WT strain were completely sensitive at the relatively low concentration of 50 mM chlorate. A similar growth response to chlorate was obtained when the strains were grown on 10 mM proline (Table 1, Fig. 2), which has been the preferred nitrogen compound as well as the concentration used in previous studies to generate *nrtA* mutants (Kinghorn *et al.*, 2005). In this regard, the relatively high concentrations of chlorate required to generate chlorate-resistant mutants (lacking nitrate reductase or the NrtA transporter; Kinghorn *et al.*, 2005) on proline as the nitrogen source probably reflects the basal level of *nrtA* transcript synthesized, and by extension NrtA protein, when strains are grown under such conditions of nitrate non-inducibility (i.e. in the absence of nitrate) and non-nitrogen metabolite repressibility (i.e. in the absence of ammonium or glutamine) (Unkles *et al.*, 2001).

**Table 1:** MICs of WT and mutant strains Strains were grown on solid AMM containing two concentrations of nitrate or proline, 1 mM or 10 mM, as sole sources of nitrogen, at 37 °C for 2–3 days. An aqueous solution of chlorate, chlorite, caesium or sulphite salts (pH 6.5) was added to the medium as described in Methods. ng, No growth observed for this strain with nitrate as sole nitrogen source except for the presence of scavenger hyphae, indicating nitrogen starvation.

				MIC (mM)			
Strain	Functional transporters	Nitrogen source	Source concn (mM)	Chlorate	Chlorite	Caesium	Sulphite
WT	NrtA, NrtB	Nitrate	1	100	0.2	100	15
*nrtA1*	NrtB		1	>200	0.2	40	15
*nrtB110*	NrtA		1	50	0.1	>100	15
T110	Neither		1	ng	ng	ng	ng
WT	NrtA, NrtB	Nitrate	10	>200	0.5	>100	15
*nrtA1*	NrtB		10	>200	0.5	30	15
*nrtB110*	NrtA		10	200	0.5	>100	15
T110	Neither		10	ng	ng	ng	ng
WT	NrtA, NrtB	Proline	1	10	0.5	80	15
*nrtA1*	NrtB		1	>200	0.5	80	15
*nrtB110*	NrtA		1	50	0.5	>100	15
T110	Neither		1	>200	>0.5	100	15
WT	NrtA, NrtB	Proline	10	25	1	60	>25
*nrtA1*	NrtB		10	>200	2	60	>25
*nrtB110*	NrtA		10	50	3	>100	>25
T110	Neither		10	>200	5	>100	>25

The kinetic results for chlorate inhibition in net nitrate transport experiments (Table 2) gave *K*_i_ values of around 44 mM for NrtB (mutant *nrtA1*), 29 mM for NrtA (mutant *nrtB110*) and 30 mM for the WT strain (containing both functional proteins), showing that NrtA activity is statistically significantly more inhibited than NrtB, according to confidence limits shown by se data. Moreover, at lower concentrations of chlorate (10 mM), mutant *nrtA1* (expressing a functional NrtB) retained 93 % net nitrate transport activity, whereas mutant *nrtB110* (expressing a functional NrtA) possessed only 65 % activity (data not shown). However, our results of nitrate transport inhibition by chlorate contradict the results of Brownlee & Arst (1983). They reported 80 % reduction of nitrate transport in the WT strain and *crnA1* (*nrtA1*) mutant by 1 mM chlorate in cells grown for 6 or 17 h. The reason for this discrepancy is unclear.

**Table 2:** Kinetic constants for nitrate transport and inhibition by the *A. nidulans* strains Strains were grown and induced as described in Methods. Also, the *K*_i_ (inhibition constant) values were determined as described in the Methods. Values of *K*_m_ (inverse measure of the substrate's affinity for the enzyme) for the WT and mutant strains were compared with their respective *K*_i_ for each inhibitor in order to identify competition of nitrate by inhibitor (if any) for individual transporters. Values of all parameters are given as the mean ± se of at least three independent experiments.

				*K*_i_ (mM)		
Strain	Functional nitrate transporter(s)	*V*_max_	*K*_m_ (μM)	Chlorate	Chlorite	Caesium
WT	NrtA, NrtB	14.2 ± 0.8	57.1 ± 2.6	29.9 ± 3.7	0.35 ± 0.02	45.1 ± 6.1
*nrtA1*	NrtB	3.6 ± 0.2	11.4 ± 2.4	44.3 ± 2.3	0.34 ± 0.02	2.6 ± 0.02
*nrtB110*	NrtA	9.1 ± 0.8	41.0 ± 4.8	29.3 ± 2.8	0.49 ± 0.01	54.8 ± 0.7

**Fig. 6.**
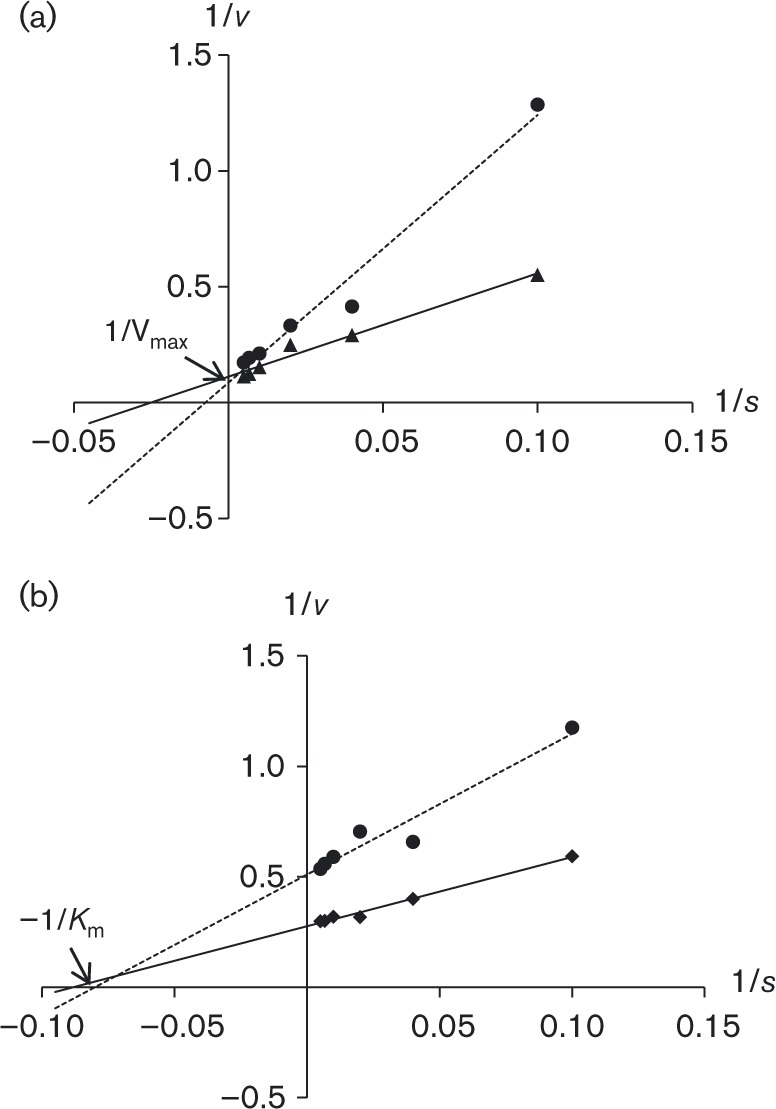
Chlorate inhibition of NrtA and NrtB transporters. Lineweaver–Burk plots showing the effect of chlorate on NrtA and NrtB at a series of nitrate concentrations. Chlorate showed competition with nitrate for NrtA (expressed in *nrtB110*) (a) and for NrtB (expressed in *nrtA1*) non-competitive inhibition by chlorate was observed (b). ♦, No inhibitor added; •, inhibitor added.

For the NrtA transporter (expressed in *nrtB110*), chlorate proved to be a competitive inhibitor, i.e. the inhibitor binds to the same site on the transporter as the natural substrate, because the *K*_m_ increased in the presence of chlorate (Fig. 6a), but the *V*_max_ was unaffected, as shown by the Lineweaver–Burk plot. By contrast, chlorate appeared to inhibit nitrate transport by the NrtB transporter non-competitively, i.e. the inhibitor affects the activity of both the empty transporter proteins and the nitrate-bound transporter proteins to inhibit the transport cycle, with similar *K*_m_ but lowered *V*_max_ in the presence of inhibitor (Fig. 6b).

### Chlorite contaminant assessment

Unlike chlorate, commercial chemical supplies of chlorite typically contain a relatively high percentage (20 %) of contaminants. To rule out the possibility that observed ‘chlorite inhibition’ was due to one of the main chemical contaminant(s), particularly chlorate, present in the chlorite product, a ‘contaminant cocktail’ solution, representing those present in the commercial supply, comprising sodium chloride, sodium chlorate, sodium hydroxide and sodium carbonate (in the ratio of 10 : 3 : 3 : 2) was prepared from individual 99 % pure grade chemicals. The pH of this solution was adjusted to 6.5 and aliquots incorporated into solid AMM (with nitrate or proline as the nitrogen source) at final concentrations equivalent to those that would be present in agar medium containing 10 mM chlorite (i.e. 1.56 mM sodium chloride, 0.28 mM sodium chlorate, 0.68 mM sodium hydroxide and 0.17 mM sodium carbonate). The chlorite contaminant cocktail concentration chosen (10 mM) is vastly (20 times) in excess of minimal chlorite inhibitory concentrations (Table 1, Fig. 3). The results (data not shown) showed no evidence of growth inhibition on plates by the ‘contamination cocktail’, thereby demonstrating that it was indeed most likely chlorite in the 80 % chlorite product that was the sole inhibitory factor.

### Chlorite inhibition studies

With solid AMM containing 1 mM nitrate as the source of nitrogen, single mutants *nrtA1* or *nrtB110* as well as the WT were completely inhibited by 0.2 mM chlorite and, with 10 mM nitrate, by 0.5 mM chlorite (Table 1, Fig. 3). With proline as the sole nitrogen source strains were more resistant to chlorite than with nitrate: at 1 mM proline all strains were completely inhibited at 0.5 mM chlorite, except for tbl110, MIC >0.5 mM. With 10 mM proline full growth inhibition occurred at 3.0 mM chlorite, except for tbl110, at 5 mM (Table 1, Fig. 3).

**Fig. 7.**
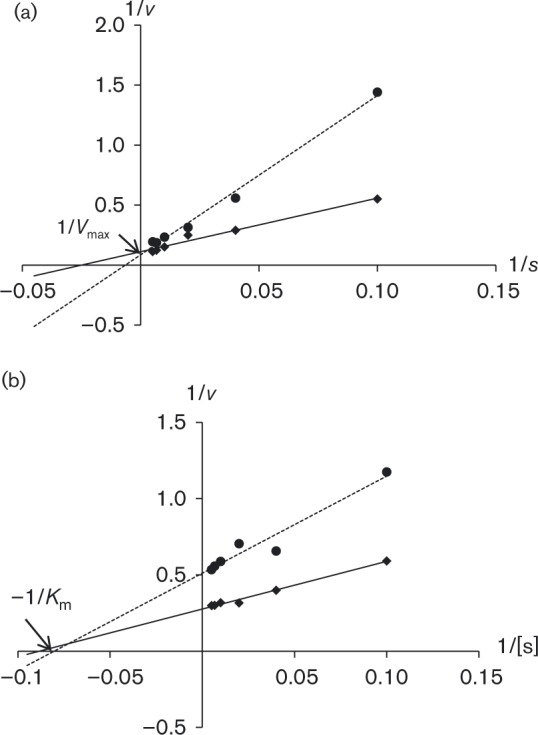
Chlorite inhibition of NrtA and NrtB transporters. Lineweaver–Burk plots showing the effect of chlorite on NrtA and NrtB at a series of nitrate concentrations. NrtA (expressed in *nrtB110*) showed competition (a) and NrtB (expressed in *nrtA1*) non-competitive inhibition (b) of nitrate uptake by chlorite. ♦, No inhibitor added; •, inhibitor added.

In contrast to the results for chlorate inhibition of nitrate uptake, extremely low concentrations of chlorite were sufficient to inhibit net nitrate transport to a substantial degree and 1 mM chlorite was sufficient to prevent transport of nitrate completely (data not shown). Compared with chlorate, kinetic analysis of inhibition by chlorite (Table 2) provided relatively low *K*_i_ values for NrtA and NrtB transporters, of 0.49 and 0.34 mM, respectively. Similar to chlorate, chlorite inhibited the nitrate uptake by NrtA in a competitive fashion (Fig. 7a), whereas inhibition of nitrate uptake by NrtB was non-competitive (Fig. 7b).

### Caesium inhibition

Caesium, a known growth inhibitor, was also tested for its toxicity/transport by the NrtA and NrtB transporters. Whereas the WT grew on 1 mM nitrate as the sole source of nitrogen in the presence of caesium concentrations up to 100 mM and mutant *nrtB110* (expressing NrtA) showed growth at the highest concentration tested, 100 mM, the *nrtA1* mutant (expressing NrtB) failed to grow on 40 mM caesium and higher concentrations. Similar results were observed with 10 mM nitrate but, in contrast, proline reversed the inhibition of the *nrtA1* mutant to some extent, doubling the MIC (Table 1, Fig. 4). The chloride ion per se at the highest concentration used had no effect on growth, as shown by the growth on 100 mM sodium chloride (Fig. 4).

From nitrate transport kinetic experiments, the *K*_i_ value for caesium calculated for mutant *nrtB110* was found to be 55 mM, and for the WT, 45 mM. In contrast, the *K*_i_ determined for the *nrtA1* mutant was approximately 3 mM, thus much lower than the WT and mutant *nrtB110* *K*_i_ values (Table 2).

**Fig. 8.**
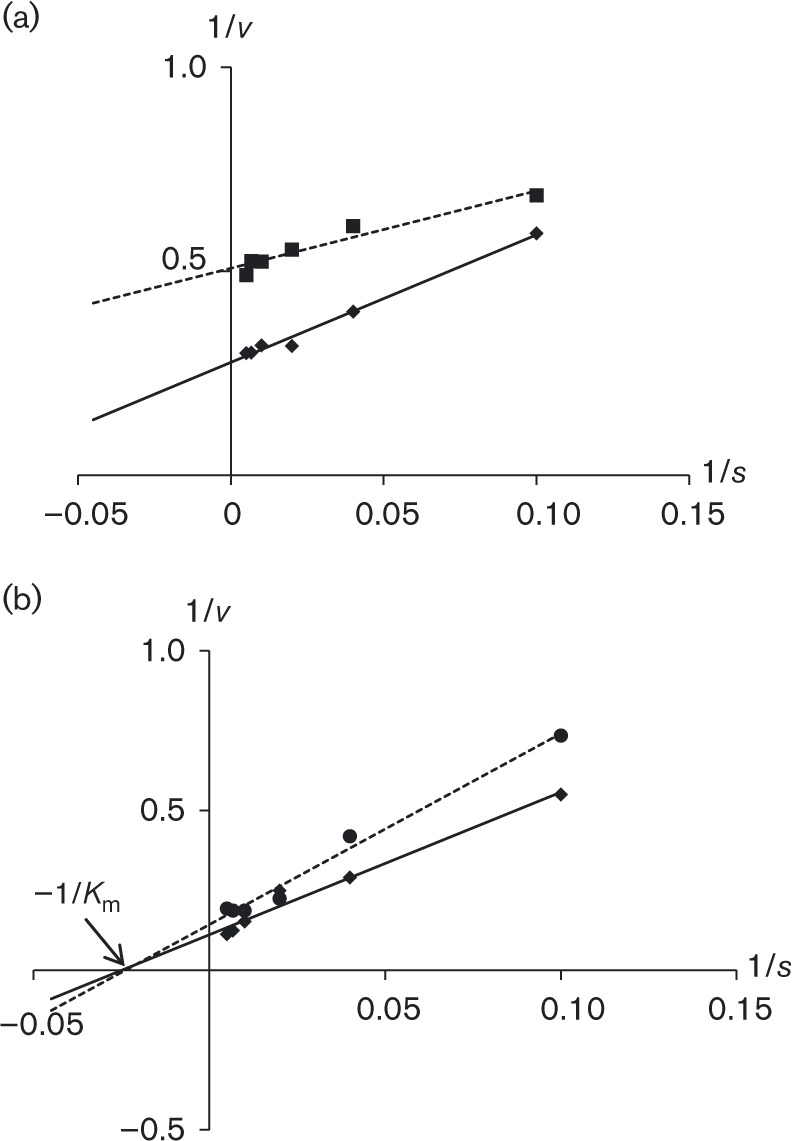
Caesium inhibition of NrtA and NrtB transporters. Lineweaver–Burk plots showing the effect of caesium on NrtA and NrtB at a series of nitrate concentrations. NrtA (expressed in *nrtB110*) showed non-competitive inhibition (a) and NrtB (expressed in *nrtA1*) uncompetitive inhibition (b). ♦, No inhibitor added; •, inhibitor added.

Caesium was recorded as a non-competitive inhibitor of nitrate uptake by the NrtA transporter because the *K*_m_ remained unchanged in the presence of caesium (Fig. 8b), resulting in the intersection of Lineweaver–Burk plot lines with the *x*-axis. The inhibition of NrtB was uncompetitive, resulting in parallel lines in the Lineweaver–Burk plot (Fig. 8a). While in non-competitive inhibition the inhibitor affects both the empty transporter proteins and the nitrate-bound transporter proteins to inhibit the transport cycle, in uncompetitive inhibition the inhibitor affects only the nitrate-bound transporter proteins to inhibit the transport cycle. In the former case the inhibitor may bind near the substrate recognition site, whereas in the latter case the inhibitor may bind elsewhere on the transporter.

### Inhibition studies with other anions

Bicarbonate, carbonic acid/bicarbonate, formate, oxalate and malonate gave no convincing phenotypic evidence of growth stunting on solid AMM, even up to relatively high concentrations (100 mM) with 1 mM nitrate (or proline) as the sole source of nitrogen (data not shown). In the case of sulphite, growth ceased at 15 mM sulphite in media that contained either 1 mM nitrate or proline. In media that contained 10 mM nitrate or proline, growth ceased at 15 or >25 mM sulphite, respectively (Table 1, Fig. 5). Sulphite toxicity was therefore less marked with growth on proline (and other nitrogen sources such as glutamate or arginine; data not shown). Net nitrate transport inhibition assay studies were carried out for these anions, except sulphite, as it was observed that rapid chemical reduction of nitrate by sulphite occurred during the time-course of these assays in the absence of fungal cells (data not shown). Additionally, the effect of carbonate, specifically available at pH 11.3, could not be assessed owing to the lack of nitrate transport at this pH. None of the remaining anions caused significant inhibition of nitrate transport (Tables 3 and 4).

**Table 3:** Assay for effects of various anions on nitrate uptake at pH 6.5

Strain	Protein	Anion	Nitrate uptake ± se (nmol NO_3_ min^− 1^ mg^− 1^)
*nrtB110*	NrtA	None	10.6 ± 1.4
		Formate	8.1 ± 0.4
		Oxalate	9.7 ± 0.4
		Carbonic acid/bicarbonate	11.1 ± 0.4
*nrtA747*	NrtB	None	2.9 ± 0.1
		Formate	2.7 ± 0.1
		Oxalate	2.4 ± 0.1
		Carbonic acid/bicarbonate	2.7 ± 0.1
		Malonate	2.7 ± 0.2

**Table 4:** Assay for effect of bicarbonate on nitrate uptake at pH 8.3

Strain	Protein	Anion	Nitrate uptake ± se (nmol NO_3_ min^− 1^ mg^− 1^)
*nrtB110*	NrtA	None	0.7 ± 0.4
		Bicarbonate	0.5 ± 0.7
*nrtA747*	NrtB	None	1.9 ± 0.7
		Bicarbonate	1.9 ± 0.3

To assess if *A. nidulans* has transport systems for potential export of nitrate similar to *H. polymorpha*, namely Nar1, Ssu1 and Ssu2 (Cabrera *et al.*, 2014), sequences of these proteins were searched against all proteins encoded by the genome of *A. nidulans*. The best match to the Nar1 sequence was that of the nitrite transporter NitA, though with only 32 % identity over a 200 aa length alignment. Nar1 is substantially longer than NitA (476 compared with 310 aa), with the main extension at the C-terminal end. For both Ssu1 and Ssu2, the top matches were to the putative malate uptake permease from *A. nidulans*, with low identities, 25 % over a 415 aa match and 46 % over a 377 aa match, respectively. In contrast, a substantially higher match was found between Ssu1from *A. fumigatus* and the hypothetical protein AN9133.2 from *A. nidulans*, exhibiting 72 % identity over a 379 aa alignment.

## Discussion

Initially, we assessed the ability of mutants expressing NrtA or NrtB individually to grow with a limiting nitrate concentration (1 mM) in the presence of various concentrations of potential inhibitory analogues of nitrate. After growth inhibition studies, kinetics of nitrate uptake were evaluated in the presence or absence of an inhibitor. Unpredictably, perhaps, chemically similar molecules, such as formate and bicarbonate, did not inhibit growth or nitrate uptake of the WT or mutants. Also, the two compounds, malonate and oxalate, that resembled two nitrate molecules joined together did not inhibit growth or nitrate uptake. So it would appear that these compounds are not substrates for uptake by NrtA or NrtB. Growth of the test strains on nitrate at different concentrations was uniformly reduced in the presence of sulphite and also on 1 mM proline, indicating a general toxicity, though 10 mM proline provided some protection. As sulphite reacted chemically with nitrate during our assay experiments, the kinetics of nitrate uptake in its presence and, hence, specificity of the transporters for sulphite could not be determined.

The effect of chlorate on growth strongly suggests that NrtA transports chlorate into *A. nidulans* cells at substantially higher rates than NrtB. Firstly, at the near growth-limiting nitrate concentration of 1 mM in solid AMM, reduced growth was exhibited by the *nrtA1* mutant (expressing the NrtB transporter) only at a chlorate concentration of 100 mM, and, even at 200 mM chlorate, growth of this mutant was appreciable. This suggested that the NrtB protein was not affected or at least was less affected by the presence of chlorate in agar medium than the WT or *nrtB110* strains. Secondly, and in contrast, mutant *nrtB110* (expressing the NrtA transporter) failed to grow in the presence of 50 mM chlorate with 1 mM nitrate, demonstrating that nitrate transport activity by the NrtA protein is markedly reduced by the presence of chlorate. As expected of a competitive inhibitor, growth inhibition on such low concentrations of chlorate was overcome in our growth experiments by increasing the concentration of the substrate nitrate to 10 mM. Thirdly, with 1 or 10 mM proline as the sole source of nitrogen and as observed in the past (Kinghorn *et al.*, 2005), *nrtA* mutants (as exemplified by *nrtA1*) grew in the presence of at least 200 mM chlorate, whilst mutant *nrtB110* and the WT failed to grow at 50 mM chlorate in both cases. Such growth responses on proline as the nitrogen source indicate that chlorate must enter the WT and *nrtB110* mutant cells to result in cellular toxicity. Moreover, the lack of chlorate toxicity in the *nrtA1* mutant strain is most likely due to the absence of NrtA activity and, by extrapolation, suggests that NrtA is the major transporter of chlorate, as it is of nitrate (Unkles *et al.*, 2001). Finally, the growth response results indicated that chlorate was a poor substrate for the NrtB transporter. The chlorate *K*_i_ values of around 44 mM for NrtB compared with 29 mM for NrtA, from our net nitrate transport assay experiments, are consistent with this notion.

The transport kinetics results suggest that NrtA does indeed transport chlorate into cells, as the inhibition is competitive for NrtA. Crystal structures of MFS proteins indicate that the alternating access mode of action of these proteins involves a single substrate-binding cavity and so the observation of competitive binding implies that chlorate is competing with nitrate for the substrate-binding residues. In contrast, for NrtB, the inhibition by chlorate is non-competitive, inferring that chlorate is not competing directly with nitrate and therefore not transported by NrtB. Instead, chlorate may simply inhibit the transport cycle of NrtB by tending to stabilize the protein structure at one point in the transport cycle.

Therefore, growth test responses taken together with transport kinetic data provide compelling evidence that chlorate is an effective substrate for the NrtA transporter. Clearly, it is on this basis that selection for nitrate transport mutants using chlorate resulted in isolating mutants defective in NrtA rather than NrtB (Cove, 1976). The high resistance of the *nrtA1* mutant (with a functional NrtB protein) to chlorate can be explained by the lack of transport of chlorate by NrtB, demonstrated by the non-competitive nature of this inhibitor for this transporter.

Growth test results suggest that chlorite is highly toxic for both single mutants, expressing NrtA or NrtB individually, as well as for the WT, and appears to affect the mutant expressing NrtA slightly more than that expressing NrtB when grown on proline, though not on nitrate. Consistent with growth tests of chlorite with nitrate, the kinetic analysis for chlorite inhibition of nitrate transport (Table 2) gave relatively low *K*_i_ values (0.34 to 0.5 mM) for both mutants and WT. The differential effect of chlorite on growth supported by nitrate compared with proline might possibly be due to different expression of NrtA and/or NrtB on these different nitrogen sources. Clearly, chlorite is a very reactive molecule and we suggest that its effects are not specific to nitrate transport, as it was also very toxic when the fungus was grown on proline, so that chlorite is most likely toxic towards a number of cellular components. Moreover, chlorite appears to be a significant substrate for the *A. nidulans* nitrite transporter, NitA (data not shown), and thus may enter cells in this way: this is consistent with the similar chemical structures of nitrite and chlorite (Fig. 1). In terms of chlorite transport by NrtA, our kinetic studies support the results of Zhou *et al.* (2000), whose electrophysiological studies of NrtA expressed in *Xenopus* oocytes suggested that chlorite was a substrate for this transporter. They did not, however, observe transport of chlorate, perhaps reflecting the low *K*_i_ determined in our study. Although the chlorite *K*_i_ values for NrtA and NrtB were fairly similar, further kinetic studies showed that nitrate uptake by NrtA was inhibited in a competitive fashion by chlorite, whereas inhibition of NrtB nitrate uptake was non-competitive. Therefore, it is possible that both chlorate and chlorite could block nitrate uptake completely by NrtA under conditions where nitrate is limiting, by competing with nitrate for the substrate-binding residues within the translocation pathway of the protein. The non-competitive inhibition observed for NrtB would suggest that neither chlorate nor chlorite enters the cell via the NrtB protein.

Inhibition of *A. nidulans nrtA* mutant growth by caesium has been established previously (Brownlee & Arst, 1983; Unkles *et al.*, 2001). In parallel with these studies, both growth tests and kinetic results indicate that NrtB nitrate transport is more strongly inhibited by the cation caesium than is NrtA. Caesium, which is toxic to biological systems, is chemically similar to potassium, an essential element. It has been previously reported that caesium can inhibit the enzymes activated by potassium (Avery, 1995) and also inhibits the cellular uptake of potassium ions (White & Broadley, 2000), resulting in potassium starvation. Hampton *et al.* (2004) demonstrated competition between potassium and caesium for potassium-binding sites on essential proteins in *Ara. thaliana.* The kinetic evidence presented here of non-competitive and uncompetitive inhibition of nitrate uptake by caesium suggests that the cation is, predictably, a substrate for neither NrtA nor NrtB, respectively. Therefore, possible reasons for differential caesium toxicity as determined by Unkles *et al.* (2001) and in this present study may include variable expression of monovalent cation uptake. Alternatively, the response of NrtA and NrtB transporters to caesium could be explained by differences in their protein sequences leading to differential caesium binding. Such binding could have indirect effects on the transport mechanism, such as stabilization of the protein structure to prevent transport of nitrate or the blocking of proton symport (energy source). For example, the large caesium cation might potentially bind to E330 in NrtA, an essential charged residue proposed to be involved in the associated proton symport (Unkles *et al.*, 2004), and perhaps more strongly to the equivalent E322 in NrtB.

Sulphite toxicity was less marked with growth on proline (and other nitrogen sources such as glutamate or arginine) compared with growth on nitrate. Perhaps this difference may be due to changes in efficiency of sulphite export. *A. fumigatus* has the capability to export sulphite by the Ssu1 transporter (Léchenne *et al.*, 2007), and its sequence shares substantial similarity with the hypothetical protein AN9133.2 encoded by *A. nidulans*. It is possible then that sulphite export can occur in *A. nidulans,* and this is inhibited to some degree by nitrate but not by proline, so that in the latter case cells are more resistant to sulphite than in the former.

The lack of inhibition of nitrate transport by various common anions (Fig. 1) for both NrtA and NrtB indicates the generally specific recognition of nitrate by both these transporters. This selective characteristic may be important for growth of *A. nidulans* under environmental conditions that include common anion analogues, such as bicarbonate. The chemical properties of inhibitors may help indicate the basis of the substrate specificity for nitrate transport. Chlorine has a similar electronegativity to nitrogen, so that chlorate is likely to contain a comparable charge separation to nitrate, with a similar δ+ value on the central Cl or N atom and similar δ −  on the three distal O atoms. The structurally similar molecules nitrite and chlorite would also exhibit comparable charge separation. These similarities, in structure and charge separation, may be the basis for substrate recognition by both NrtA and NrtB: NrtA appears to transport nitrate, chlorate and chlorite. In contrast, the lack of inhibition of nitrate transport by the structurally similar bicarbonate molecule may be due to the lower electronegativity of carbon, giving a substantially greater charge separation between the central C atom and the three distal O atoms. That is, the NrtA and NrtB transporters may selectively recognize nitrate owing to its intermediate polarity, compared with compounds with similar structure but higher polarity. The efficient transport of nitrite by both NrtA and NrtB, reported by Wang *et al.* (2008), is consistent with the proposed recognition of moderate charge separation by the transport mechanism in these transporters. Also consistent are the conserved polar residues, such as R87 and R368 in NrtA, which are essential for efficient transport (Unkles *et al.*, 2004, 2012), and thus might be involved in selectively recognizing substrates owing to moderate charge separation in these molecules. Also, central atom size in the substrate appears not to be over-critical, given that C has a van der Waals volume between that of N and Cl (C, 20.58 Å^3^; N, 15.60 Å^3^; Cl, 22.45 Å^3^), and bicarbonate appeared not to inhibit nitrate transport, while chlorate markedly reduced transport of nitrate. Molecular shape differences, trigonal pyramid (chlorate) compared with trigonal planar (nitrate), do not appear to be a significant factor in substrate selection. Distinct from the polar nitrate/nitrite-binding sites of NrtA, NrtB, NarK and NarU, which coordinate the substrate with two opposing conserved Arg residues, the nitrate-binding pocket in *Ara. thaliana* NRT1.1 is predominantly formed by hydrophobic residues. In this case a polar residue present, H356, has been proposed to interact with nitrate and has been shown to be essential for transport (Sun *et al.*, 2014). Whether this transporter provides different substrate selection compared with NrtA and NrtB in *A. nidulans* is unknown.

It is apparent that *A. nidulans* NrtA and NrtB, which are significantly disparate in their protein sequences, are also dissimilar in their physiology, as demonstrated by their differential responses to inhibition by chlorate, chlorite and caesium, as well as different affinities for nitrate (and nitrite; Wang *et al.*, 2008). Nevertheless, both transporters substantially select nitrate in preference to structurally similar common anions, which may be important for survival and growth of *A. nidulans* in natural environments.

Finally, *A. nidulans* appears not to have versions of the nitrate export transporters identified in *H. polymorpha*, Nar1, Ssu1 and Ssu2, with only weak matches to two transporters in *A. nidulans*, including the NitA nitrite transporter. However, the significant match found between AN9133 from *A. nidulans* and Ssu1 from *A. fumigatus* indicates a need to investigate the role of AN9133. In addition, homologues of the POT, such as *Ara. thaliana* Nrt1.1, in *A. nidulans*, AN3408, AN8903 and AN1073, do not possess the residues that are crucial for coordinating nitrate (Parker & Newstead, 2014) and so are very unlikely to transport nitrate as a substrate. Thus, for *A. nidulans*, and likely other filamentous fungi, it appears that NrtA and possibly NrtB have the main roles in exporting nitrate, and NrtA, NitA and possibly NrtB for export of nitrite, when required under certain conditions (Wang *et al.*, 2007, 2008).
